# Cisplatin - induced peripheral neuropathy and biomarkers of gut microbial translocation in testicular germ cell tumor survivors

**DOI:** 10.3389/fimmu.2026.1827640

**Published:** 2026-05-11

**Authors:** Dominika Rychtarikova, Katarina Kalavska, Jana Obertova, Patrik Palacka, Katarina Rejlekova, Zuzana Sycova-Mila, Zuzana Orszaghova, Peter Lesko, Rateb Alzeer, Lucia Vasilkova, Daniela Svetlovska, Beata Mladosievicova, Michal Pastorek, Matej Rychtarik, Barbora Vlkova, Peter Celec, Michal Mego, Michal Chovanec

**Affiliations:** 1 2nd Department of Oncology, Comenius University, Faculty of Medicine &amp, National Cancer Institute, Bratislava, Slovakia; 2 Translational Research Unit, 2nd Department of Oncology, Comenius University, Faculty of Medicine &amp, National Cancer Institute, Bratislava, Slovakia; 3 Department of Medical Oncology, National Cancer Institute, Bratislava, Slovakia; 4 Department of Psychology, Faculty of Philosophy, Comenius University, Bratislava, Slovakia; 5 Department of Clinical Trials, National Cancer Institute, Bratislava, Slovakia; 6 Institute of Pathological Physiology, Comenius University, Faculty of Medicine, Bratislava, Slovakia; 7 Faculty of Medicine, Institute of Molecular Biomedicine, Comenius University, Bratislava, Slovakia; 8 Biomedical Research Center, Slovak Academy of Sciences, Cancer Research Institute, Bratislava, Slovakia

**Keywords:** biomarker, cancer survivors, cancer treatment, cisplatin induced peripheral neuropathy, gut microbial translocation, late toxicity, testicular germ cell tumor

## Abstract

**Background:**

Cisplatin-induced peripheral neuropathy (CIPN) is a frequent and often persistent complication in survivors of testicular germ cell tumors (GCT) treated with curative therapy. Although several mechanisms have been proposed, the biological drivers of long-term neurotoxicity remain incompletely understood. Disruption of intestinal barrier integrity during chemotherapy or radiotherapy may promote gut microbial translocation (GMT), leading to systemic immune activation and chronic inflammation that could contribute to neuropathy development. This study investigated the relationship between circulating biomarkers of GMT and symptoms of CIPN in long-term GCT survivors.

**Methods:**

A total of 170 GCT survivors (median age 41 years) from the National Cancer Institute of Slovakia were included, with a median follow-up of 10 years after treatment. Participants completed the EORTC QLQ-CIPN20 questionnaire assessing sensory, motor, and autonomic neuropathy. Peripheral blood samples were analyzed for plasma biomarkers associated with gut microbial translocation and innate immune activation, including soluble CD14 (sCD14), high-mobility group box-1 (HMGB1), lipopolysaccharide (LPS), and D-lactate. Associations between biomarker concentrations and CIPN scores were evaluated across treatment groups: orchiectomy only (active surveillance, n=28), cisplatin-based chemotherapy (n=119), radiotherapy (n=14), and combined chemoradiotherapy (n=9).

**Results:**

Patients with higher plasma sCD14 levels had significantly higher overall CIPN scores (7.6% increase, p=0.019) and worse sensory function (9.5% increase, p=0.019) compared with those with lower levels. Patients treated with chemotherapy exhibited significantly higher plasma sCD14 levels than those under active surveillance (6613 vs. 3768 μg/L, p = 0.009). Among chemotherapy-treated patients, elevated sCD14 was associated with a higher risk of motor neuropathy (RR = 3.5, 95% CI 1.21–10.14, p=0.020). In survivors receiving combined chemotherapy and radiotherapy, increased sCD14 levels were associated with a higher risk of autonomic neuropathy (RR = 2.85, 95% CI 1.11–7.30, p=0.029). Elevated HMGB1 was also associated with an increased probability of autonomic dysfunction (RR = 2.15, 95% CI 1.07–4.33, p=0.015). No significant associations were observed between cumulative cisplatin dose and GMT biomarkers.

**Conclusion:**

Elevated biomarkers of gut microbial translocation, particularly sCD14, are associated with increased severity of CIPN in long-term GCT survivors. These findings support the hypothesis that treatment-related intestinal barrier disruption and subsequent immune activation may contribute to persistent neurotoxicity in cancer survivorship.

## Introduction

Germ cell tumors (GCTs) represent the most frequent malignancy among young adult males, typically occurring between the ages of 15 and 40 (1). Due to the high chemosensitivity to cisplatin-based regimens, GCTs are considered the most curable solid tumors, even in the metastatic setting (2). Around 90% of patients with metastatic GCT can be cured (3, 4). Despite excellent survival outcomes, long-term toxicities of treatment remain a major concern (5). Cisplatin-induced peripheral neuropathy (CIPN) represents one of the most common and persistent complications, with recent evidence indicating that approximately 47.8% of patients with CIPN experience chronic moderate-to-severe or painful neuropathy lasting at least three months after treatment (6). Cisplatin, a cornerstone of GCT chemotherapy, is well known for its neurotoxic potential. Cisplatin-induced peripheral neuropathy most commonly presents as a chronic, predominantly sensory peripheral neuropathy. In addition to peripheral nervous system effects, accumulating evidence suggests that cisplatin may also contribute to central neurotoxicity, including cognitive dysfunction. These neurological sequelae can persist for years after treatment completion and may significantly impair survivors’ quality of life. Although CIPN is a well-recognized clinical condition in GCT survivors, its underlying mechanisms remain poorly understood. Multiple processes—such as direct neuronal injury, mitochondrial dysfunction, oxidative stress, neuroinflammation, and impaired axonal transport—have been proposed to contribute to its development, but no single unifying mechanism has been established (2, 7, 8).

Based on our previous findings, we have focused on the late toxicities of cisplatin, particularly its effects on cognitive and neurological function. Building on this work, the present study investigates the hypothesis that alterations in the gut microbiome may contribute not only to cognitive dysfunction, but also to the development of peripheral neuropathy—one of the most debilitating long-term consequences of cisplatin-based chemotherapy.

Chemotherapy and radiotherapy used in the treatment of GCT survivors are known to impair intestinal barrier integrity, leading to the systemic dissemination of microbial products (9, 10). This microbial translocation may promote the activation of proinflammatory pathways, which are contributing to CIPN (11). Several biomarkers reflecting microbial translocation and innate immune activation have been well characterized. Among these, soluble CD14 (sCD14), lipopolysaccharide (LPS), high-mobility group box 1 (HMGB-1), and D-lactate are widely recognized as indicators of gut microbial translocation (GMT) (5).

Based on this rationale, we hypothesized that increased gut microbial translocation, reflected by elevated circulating levels of sCD14, HMGB1, LPS, and D-lactate, is associated with the presence and severity of CIPN in long-term GCT survivors. The objective of the present study was to assess circulating levels of these GMT-related biomarkers in a cohort of testicular germ cell tumor survivors and to investigate their potential relationship with CIPN.

## Patients and methods

**Figure 1:**
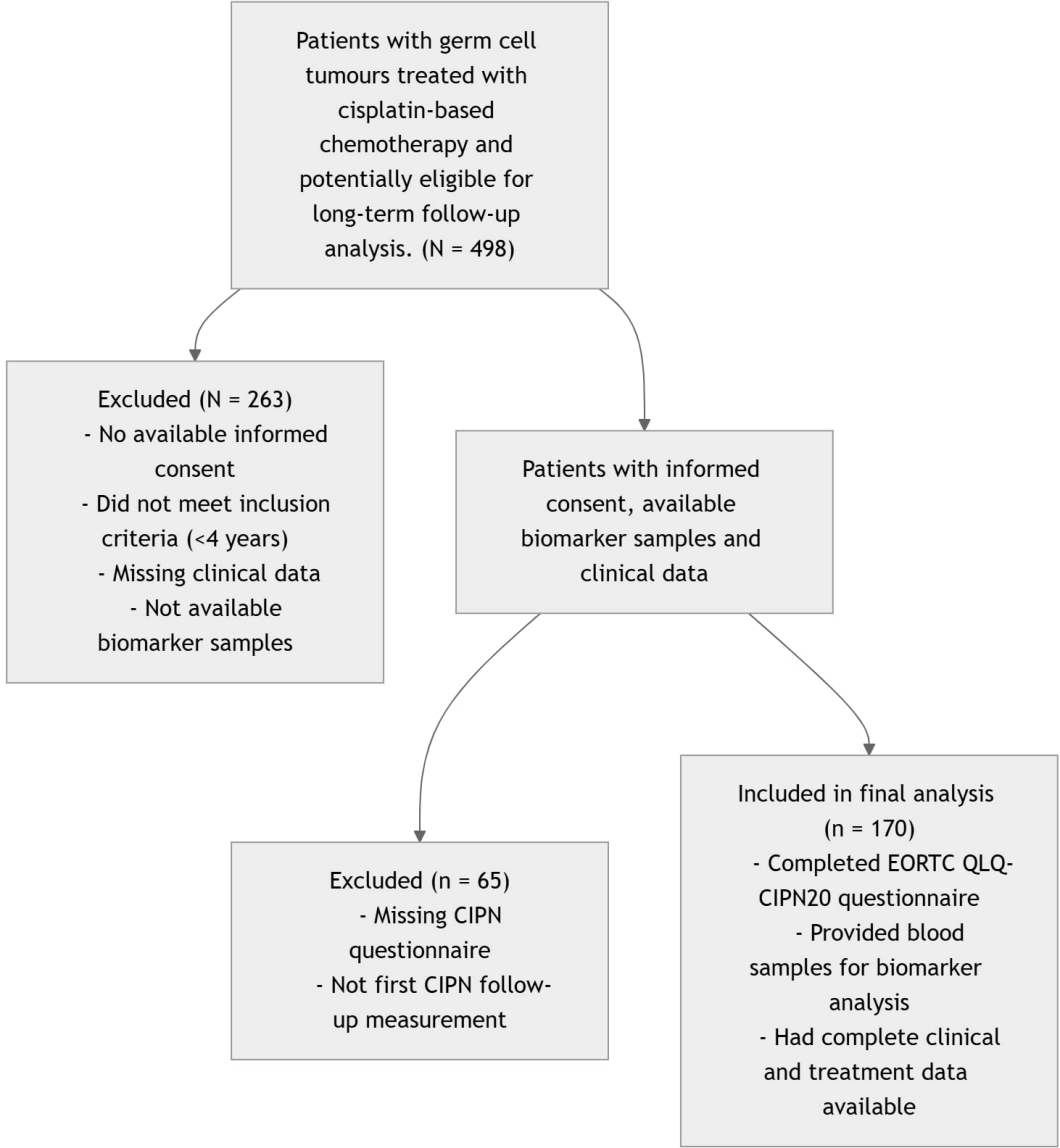
Flow diagram of participant inclusion and analysis.

This study was conducted as a component of an ongoing prospective translational study (Protocol IZLO-1) evaluating long-term toxicities and their underlying mechanisms in testicular GCT survivors. The present analysis represents a cross-sectional evaluation of selected biomarkers of GMT and their association with CIPN. Here, we assessed the selected biomarkers of GMT and their associations with CIPN in survivors who had received chemotherapy and/or radiotherapy as their curative treatment for GCT. This study included patients treated for testicular GCT at the National Cancer Institute in Slovakia between 1986 and 2015 who participated in the institution’s established annual follow-up program. Long-term surveillance of GCT survivors is conducted in accordance with institutional guidelines as part of the testicular cancer survivorship protocol. Only individuals with a minimum follow-up of four years after treatment completion were eligible for inclusion. A notable number of patients treated during this period were lost to follow-up; however, the extent of this attrition and the underlying reasons for non-compliance remain unknown. As a result, the study cohort consists solely of survivors who adhered to the follow-up protocol. A flow diagram illustrating patient inclusion, exclusion, and final study cohorts is provided in **Figure 1**.

Patients were assigned to treatment groups according to their completed therapeutic regimens.

The groups comprised orchiectomy only (AS – active surveillance, serving as our control group), radiotherapy (RT), chemotherapy (CT), and combined chemotherapy and radiotherapy (CTRT). Within the chemotherapy-treated cohort, patients were further stratified by cumulative cisplatin dose (<400 mg/m^2^ vs. ≥400 mg/m^2^), with additional comparisons made against the control group to evaluate the effect of treatment intensity. Precise cumulative dose values were not available, and therefore dose exposure was analyzed using predefined categories. The dataset analyzed in this study was constructed using data obtained from 170 patients, including peripheral blood samples and responses to the CIPN20 questionnaire.

This study received approval from the Institutional Review Board of the Slovak National Cancer Institute Ethics Committee in Bratislava. All participants provided written informed consent prior to inclusion. Enrollment occurred between September 2015 and April 2017.

### Study measures

Testicular GCT survivors attended annual follow-up visits at the 2nd Department of Oncology, Comenius University, National Cancer Institute in Bratislava, Slovakia. During these visits, participants completed the EORTC QLQ – CIPN20 in a dedicated, quiet office designed to provide a consistent environment for all participants. Survivors completed the questionnaire independently as a patient-reported measure of neuropathy symptoms, with a study nurse available nearby to offer assistance if requested.

At each follow-up, clinical data—including age, initial histological diagnosis, treatment details, and physical examination findings—were collected alongside the evaluation of neurotoxicity. The EORTC CIPN20 questionnaire is a tool designed to assess chemotherapy-induced neurotoxicity, specifically peripheral neuropathy in cancer patients. It consists of 20 items divided into three domains: sensory (9 items), motor (8 items), and autonomic (3 items). Each item is rated on a 4-point Likert scale, where higher scores indicate greater symptom severity (12).

### Study outcomes

The primary outcome of this study was the presence and severity of CIPN, assessed using the EORTC QLQ-CIPN20 questionnaire, as previously described. Secondary outcomes included circulating levels of GMT - related biomarkers, specifically soluble sCD14, LPS, HMGB1 and D-lactate. The association between CIPN severity and GMT biomarkers was evaluated as an exploratory objective.

### Evaluation of biomarkers of gut microbial translocation

Peripheral blood samples were collected from all participants on the day of their annual follow-up visit as part of this translational study (median time since treatment: 10 years; range: 4–32 years). During the same visit, participants completed the EORTC QLQ – CIPN20 questionnaire to evaluate self-reported neuropathy.

Plasma concentrations of sCD14, HMGB-, LPS, and D-lactate were measured to assess systemic inflammation, microbial translocation, and gut barrier integrity.

Peripheral blood samples were collected into BD Vacutainer^®^ K2EDTA Tubes (BD Biosciences, Franklin Lakes, NJ, USA) containing ethylenediaminetetraacetic acid in the morning of the annual follow-up visit (n=180). Blood samples (1 ml) were initially centrifuged at 2, 500 × g for 10 min and subsequently at 3, 500 × g for 10 min to separate the plasma from the blood cells. Plasma aliquots were stored at −80 °C until further analysis. HMGB1 (LS-F4038; LSBio, Seattle, WA, USA), LPS (abx150357; Abbexa, Cambridge, United Kingdom) and sCD14 (HK320-02; Hycult Biotech, Uden, Holland) were all measured using commercially available ELISA kits and the corresponding calibration standards. D-lactate was assessed using an enzymatic colorimetric assay (MAK058; Sigma-Aldrich, St. Louis, MO, USA). Intra-assay and inter-assay coefficients of variation for all assays were below 5% and 10%, respectively.

### Statistical analysis

The characteristics of patients were categorized and summarized in **Table 1**. To compare continuous variables between treatment groups, t-test was applied. For data that were not normally distributed, a nonparametric Kruskal-Wallis test was used to examine the associations between CIPN and biomarkers of GMT. The nonparametric Mann–Whitney U test was used to assess the association between the treatment modality and levels of GMT biomarkers, as well as the association between the cumulative dose of cisplatin and GMT biomarker levels. The median follow-up time was defined as the median duration from the last administered treatment in all GCT survivors. Statistical significance was determined with a p-value threshold of <0.05, and all p-values were two-sided. No formal correction for multiple comparisons was applied, as the analyses were exploratory. Therefore, results should be interpreted with caution and require validation in independent cohorts. Statistical analysis was performed using NCSS 10 (2015; Hintze J, Kaysville, Utah, USA) and Python 3.12.6. Data preprocessing was conducted in Python using the pandas library (version 2.2.0), including data cleaning, median calculation, and variable dichotomization, after which the processed datasets were exported to NCSS for statistical analysis.

**Table 1:** Clinical characteristics of patients.

Characteristic	Category	N (n=170)	%	AS	CT	RT	CTRT	p value
Age (years)								
Median (range)	41 (23 - 64)							
Follow up (years)								
Median (range)	10 (4 - 32)							
Histology	Pure seminoma	81	47,65	19	40	13	9	0,001
	Non - seminoma/mixed GCT	80	47,06	8	71	1	0	
	Histology unknown	9	5,29	1	8	0	0	
Primary tumor	Gonadal	162	95,29	28	111	14	9	0,353
	Primary retroperitoneal	7	4, 12	0	7	0	0	
	Primary mediastinal	1	0,59	0	1	0	0	
IGCCCG risk group	Good risk	122	71,76	25	76	12	9	0,750
	Intermediate risk	17	10	0	17	0	0	
	Poor risk	23	13,53	0	22	1	0	
Initial stage	I	6	3,53	3	1	1	1	0,000
	IS - III.A	66	38, 81	0	60	0	6	
	III.B	17	10	0	17	0	0	
	III.C	23	13,53	0	22	1	0	
	Unknown	13	7,65	2	8	2	1	
Treatment	AS	28	16.47					0.186
	RT only	14	8, 24					
	CT only	119	70					
	1st line only	103	60, 59					
	more than 1st line	16	9, 41					
	CTRT	9	5, 3					
Initial chemotherapy	3xBEP	45	26,47	0	45	0	0	0,000
	4xBEP	36	21,18	0	35	0	1	
	4xEP	19	11,18	0	13	0	6	
	other	35	20, 59	0	25	6	4	
Post - chemotherapy RPLND	No	129	75,88	23	86	13	7	0,676
	Yes	30	17,65	0	28	0	2	
	Unknown	11	6,47	5	5	1	0	
Time from the end of treatment	4 - 10	101	59, 42	28	64	5	4	0,167
	11 - 15	41	24, 12	0	31	7	3	
	>15	28	16, 47	0	24	2	2	

GCT, germ cell tumor; AS, active surveillance; CT, chemotherapy; RT, radiotherapy; CTRT, chemotherapy and radiotherapy; IGCCCG, International Germ Cell Cancer Collaborative Group; BEP, bleomycin, etoposide, cisplatin; EP, etoposide, cisplatin; RPLND, retroperitoneal lymph node dissection.

## Results

### Characteristics of patients

Survivor population in the study consisted of 170 GCT survivors with median age of 41 years at the follow-up (range: 23–64 years). The median follow-up was 10 years (range: 4–32 years). Characteristics of patients are shown in **Table 1**. Patients with seminoma and those with non-seminoma were represented in nearly equal numbers. The majority of patients had a primarily gonadal localized tumor and a good prognosis according to the IGCCCG classification. One patient in our cohort did not have a GCT but rather a non-germ cell testicular tumor. However, we decided to include this patient as the treatment approach and follow-up were comparable to those of GCT patients, and he may similarly experience long-term effects of oncological treatment. Given that this represents a single case, a sensitivity analysis excluding this patient was not performed, as its impact on the overall results is likely negligible.

### Biomarkers of GMT in association with CIPN

Biomarker measurements and CIPN assessment were performed at the same follow-up visit, allowing for cross-sectional evaluation of their associations.

Scores obtained from individual domains of EORTC QLQ-CIPN20 questionnaire were analyzed in association with plasma concentrations of sCD14 (μg/L), HMGB-1(ng/L), LPS (μg/L) and d-lactate (μmol/L).

Patients with higher plasma sCD14 had a 7.6% higher overall CIPN score and a 9.5% worse sensory function compared to those with lower sCD14, with both the overall and sensory domain mean scores being significantly elevated in patients with plasma sCD14 above the median (overall CIPN, p = 0.019; sensory domain, p = 0.019).

Patients treated with chemotherapy exhibited significantly elevated plasma sCD14, approximately 76% higher than in those under active surveillance (p = 0.009) and those with higher plasma sCD14 had an increased risk of sensory dysfunction (RR = 7.31, CI = 1.06–50.47, p = 0.04). Although this association was statistically significant, the wide confidence interval indicates limited precision and suggests that the magnitude of the effect should be interpreted cautiously in terms of clinical translation. Nevertheless, the direction of the association remains consistent and may be relevant for hypothesis generation in future studies. Importantly, among chemotherapy-treated patients, elevated concentration of sCD14 was associated with an increased risk of developing neuropathy (RR = 9.06, CI = 1.32–62.10, p = 0.023), particularly motor neuropathy (RR = 3.5, 95% CI = 1.21–10.14, p = 0.02) compared to AS group.

In patients receiving combined chemotherapy and radiotherapy, elevated concentration of sCD14 was associated with an almost threefold increased risk of autonomic neuropathy (RR = 2.85, CI = 1.11–7.3, p = 0.029) and, together with D-lactate (RR = 7.2, CI = 0.96–53.65, p = 0.05), with increased risk of overall neuropathy, while sCD14 was also linked to sensory neuropathy, compared to AS group. Additionally, HMGB1 appears to serve as a stable biomarker, as elevated levels were associated with higher probability of autonomic (RR = 2.15, CI = 1.07–4.33, p = 0.015) and sensory neuropathy (RR = 5, CI = 1.11–22.82, p = 0.04) compared to AS group. In several of these associations, confidence intervals were wide, indicating limited precision and suggesting that while statistically significant, the exact magnitude of effects should be interpreted with caution, particularly in the context of clinical applicability and future validation studies.

Nevertheless, there was no difference between the groups receiving a cisplatin dose of < 400 mg/m^2^ and > 400 mg/m^2^ (**Table 2**).

**Table 2:** Gut microbial translocation biomarkers in relation to cumulative dose of cisplatin.

Cumulative dose	GMT biomarker	N	Mean	Median	SEM	p value
	sCD14 (μg/L)	N	Mean	Median	SEM	p value
0		24	4297, 88	3636, 5	521, 56	0, 208
> 400 mg/m2		69	6801, 77	4050	1005, 42	
	LPS (μg/L)	N	Mean	Median	SEM	p value
0		24	42, 21	38, 85	4, 32	0, 102
> 400 mg/m2		69	34, 29	30, 4	2, 02	
	HMGB1 (ng/L)	N	Mean	Median	SEM	p value
0		24	17254, 79	16485, 5	711, 00	0, 635
> 400 mg/m2		69	19478, 90	16762	1602, 89	
	d lactate (μmol/L)	N	Mean	Median	SEM	p value
0		23	45, 35	43, 3	5, 06	0, 120
> 400 mg/m2		65	39, 97	33, 2	2, 75	

GMT, gut microbial translocation; HMGB1, high mobility group box 1; sCD14, soluble CD14; LPS, lipopolysaccharide; SEM, standard error of the mean.

**Figure 2:**
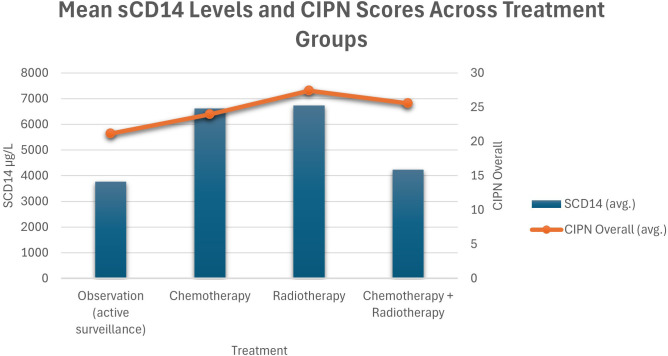
Mean sCD14 levels and CIPN scores across treatment groups (active surveillance, chemotherapy, radiotherapy, and combined chemotherapy and radiotherapy). CIPN overall scores are presented as dichotomized variables (low vs. high).

**Figure 3:**
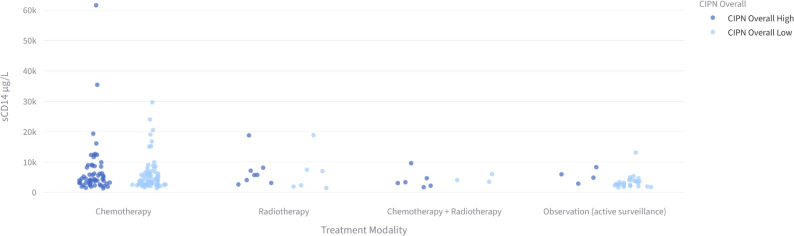
Plasma sCD14 concentrations and neuropathy status across treatment modalities.

Among radiotherapy-treated patients, those with elevated concentrations of sCD14 or HMGB1 exhibited an increased risk of overall neuropathy (sCD14: RR = 12.67, CI = 1.78–90.18, p = 0.01; HMGB1: RR = 5.63, CI = 1.46–21.72, p = 0.01) compared to AS group. Higher concentrations od sCD14 and HMGB1 were also associated with sensory neuropathy (sCD14: RR = 10.56, CI = 1.44–77.62, p = 0.02; HMGB1: RR = 4.69, CI = 1.16–18.96, p = 0.03), and elevated sCD14 was linked to an increased risk of motor neuropathy (RR = 3.52, CI = 1.07–11.58, p = 0.04) compared to AS group. Given that only a small proportion of patients (approximately 8%) received radiotherapy, these subgroup findings should be interpreted as exploratory, and the wide confidence intervals further suggest limited precision, supporting the need for confirmation in larger cohorts (**Tables 3**, **4**). These results are further illustrated in **Figures 2**, **3**, showing sCD14 levels in relation to CIPN scores and neuropathy status across treatment groups.

**Table 3:** Markers of gut microbial translocation in relation to chemotherapy-induced peripheral neuropathy.

CIPN score	GMT biomarker	N	Mean	Median	SEM	p value
	d - lactate (μmol/L)	N	Mean	Median	SEM	p value
CIPN overall	high	81	24,10	22,00	0,67	0,750
	low	89	23,66	22,00	0,57	
CIPN sensory	high	81	10,93	10,00	0,36	0,758
	low	89	11,00	10,00	0,34	
CIPN motor	high	81	9,33	9,00	0,23	0,123
	low	89	8,97	8,00	0,20	
CIPN autonomy	high	81	3,84	3,00	0,18	0,970
	low	89	3,70	3,00	0,11	
	HMGB1 (ng/L)	N	Mean	Median	SEM	p value
CIPN overall	high	76	24,47	22,00	0,71	0,186
	low	94	23,38	22,00	0,53	
CIPN sensory	high	76	11,36	10,00	0,42	0,362
	low	94	10,65	10,00	0,28	
CIPN motor	high	76	9,29	9,00	0,24	0,291
	low	94	9,02	8,00	0,20	
CIPN autonomy	high	76	3,83	3,00	0,16	0,396
	low	94	3,71	3,00	0,13	
	sCD14 (μg/L)	N	Mean	Median	SEM	p value
CIPN overall	high	87	24, 72	22, 00	0, 67	0, 019
	low	83	22,98	21,00	0,53	
CIPN sensory	high	87	11,45	10,00	0,39	0, 019
	low	83	10,46	9,00	0,29	
CIPN motor	high	87	9,39	9,00	0,23	0,087
	low	83	8,88	8,00	0,20	
CIPN autonomy	high	87	3,89	3,00	0,15	0,203
	low	83	3,64	3,00	0,14	
	LPS (μg/L)	N	Mean	Median	SEM	p value
CIPN overall	high	89	23,69	22,00	0,60	0,223
	low	81	24,07	22,00	0,63	
CIPN sensory	high	89	10,98	10,00	0,36	0,440
	low	81	10,95	10,00	0,34	
CIPN motor	high	89	9,10	8,00	0,21	0,591
	low	81	9,19	9,00	0,22	
CIPN autonomy	high	89	3,61	3,00	0,12	0,072
	low	81	3,94	4,00	0,17	

CIPN, chemotherapy-induced peripheral neuropathy; GMT, gut microbial translocation; HMGB1, high mobility group box 1; sCD14, soluble CD14; LPS, lipopolysaccharide; SEM, standard error of the mean.

**Table 4:** Gut microbial translocation biomarkers in relation to type of oncologic therapy.

Therapy modality	GMT biomarker	N	Mean	Median	SEM	p value
	sCD14 (μg/L)	N	Mean	Median	SEM	p value
AS		28	3768,36	3161	447,95	0, 009
CT		119	6613,29	4214	690,40	
	LPS (μg/L)	N	Mean	Median	SEM	p value
AS		28	39,49	33,15	3,93	0,205
CT		119	34,92	30	1,88	
	HMGB1 (ng/L)	N	Mean	Median	SEM	p value
AS		28	18591,04	17507,5	899,20	0,646
CT		119	19394,74	17387	1184,69	
	d lactate (μmol/L)	N	Mean	Median	SEM	p value
AS		27	42,91	40,3	4,50	0,250
CT		113	38,94	35,5	1,92	
	sCD14 (μg/L)	N	Mean	Median	SEM	p value
AS		28	3768,36	3161	447,95	0,081
RT		14	6729,93	5728	1489,99	
	LPS (μg/L)	N	Mean	Median	SEM	p value
AS		28	39,49	33,15	3,93	0,304
RT		14	38,51	25,5	7,87	
	HMGB1 (ng/L)	N	Mean	Median	SEM	p value
AS		28	18591,04	17507,5	899,20	0,557
RT		14	20137,93	19069,5	1557,14	
	d lactate (μmol/L)	N	Mean	Median	SEM	p value
AS		27	42,91	40,3	4,50	0,500
RT		14	38,40	31,15	4,97	
	sCD14 (μg/L)	N	Mean	Median	SEM	p value
AS		28	3768,36	3161	447,95	0,536
CTRT		9	4237,00	3500	793,14	
	LPS (μg/L)	N	Mean	Median	SEM	p value
AS		28	39,49	33,15	3,93	0,901
CTRT		9	37,86	40,2	5,37	
	HMGB1 (ng/L)	N	Mean	Median	SEM	p value
AS		28	18591,04	17507,5	899,20	0,512
CTRT		9	17860,44	15429	1606,99	
	d lactate (μmol/L)	N	Mean	Median	SEM	p value
AS		27	42,91	40,3	4,50	0,844
CTRT		8	49, 43	39, 7	9, 91	

AS, active surveillance; CT, chemotherapy; RT, radiotherapy; CTRT, chemotherapy and radiotherapy; SEM, standard error of the mean; GMT, gut microbial translocation; HMGB1, high mobility group box 1; sCD14, soluble CD14; LPS, lipopolysaccharide.

## Discussion

CIPN is a common and dose-limiting side effect of cisplatin, a platinum-based chemotherapeutic agent widely used to treat patients with testicular cancer. The development of CIPN significantly impacts quality of life of patients and may necessitate reduction or discontinuation of treatment (2). However, in the context of testicular cancer—a malignancy with one of the highest cure rates among solid tumors—treatment discontinuation or dose reduction is not a permissible compromise. In this curative setting, toxicity must be sacrificed in favor of survival, as full-dose cisplatin-based chemotherapy is essential to achieve cure. Any deviation from the optimal dose intensity risks undermining the treatment intent. Therefore, while toxicity management is crucial, it must be carefully balanced to prevent overtreatment without jeopardizing the chance of cure for the patient. When combined with radiotherapy, the risk and severity of CIPN may increase (2, 13). Patients suffering from CIPN typically experience numbness and tingling (paresthesia), burning or shooting pain, sensory loss, especially in the hands and feet and motor weakness. These symptoms of CIPN can persist long after treatment ends and may become irreversible (14).

In recent years, the gut microbiome has garnered increasing attention in modern medicine, largely due to its profound impact on human health, particularly in relation to cancer. The gut microbiome refers to the complex community of microorganisms. These microbes play a crucial role in various physiological processes, ranging from metabolism and immune regulation to maintaining the integrity of the intestinal barrier. Cancer treatments such as chemotherapy and radiotherapy can significantly disrupt this delicate microbial balance, leading to what is known as dysbiosis (an imbalance of the microbial community). This disruption can compromise intestinal barrier function and promote GMT, whereby microbial products cross the intestinal mucosa into systemic circulation. Such processes have been implicated not only in gastrointestinal toxicity, but also in systemic inflammatory responses that may contribute to late-onset treatment-related complications. Building upon this mechanistic framework, previous analyses have suggested that GMT biomarkers may be associated with symptoms of late toxicity following cancer treatment (10). Previous analyses have already suggested that gut GMT biomarkers may play a role in certain symptoms of late-onset toxicity following cancer treatment. An established association between GMT biomarkers and cognitive dysfunction, as shown in our previous research, supports this notion (15). The present study, which focuses on the relationship between GMT biomarkers and CIPN, further reinforces our hypothesis that late effects of cancer therapy may, in part, be driven by the leakage of microbial products from the gut. We observed that patients treated with chemotherapy exhibited elevated levels of sCD14. Based on our findings, patients in our cohort who were treated with chemotherapy or a combination of chemotherapy and radiotherapy and exhibited elevated levels of GMT biomarkers (specifically sCD14 and HMGB1 in our study) were at increased risk of developing neuropathy. However, given the wide confidence intervals observed in several analyses, the magnitude of these associations should be interpreted with caution. Larger, well-powered studies will be required to obtain more precise and reliable estimates. Notably, we did not observe any significant association between cumulative cisplatin dose and levels of GMT-related biomarkers. This suggests that gut microbial translocation may not be primarily driven by treatment intensity alone. We therefore hypothesize that cisplatin-induced damage may act as an initial trigger of gut barrier disruption, while the extent and persistence of GMT may be influenced by additional factors such as individual susceptibility or long-term alterations in intestinal barrier function.

As mentioned earlier, chemotherapy (especially with agents like cisplatin) and radiotherapy to the retroperitoneum can damage the gastrointestinal epithelium. This results in loss of epithelial integrity, disruption of tight junctions, increased intestinal permeability. This allows microbial products and even live bacteria to translocate across the gut barrier into systemic circulation (8). Microbial translocation triggers an immune response by activating of Toll-like receptors (TLRs) on immune cells and neurons, releasing of pro-inflammatory cytokines (e.g., TNF-α, IL-6, IL-1β), systemic and neuroinflammation, which can sensitize peripheral nerves and promote neuropathic pain (9). Chronic low-grade inflammation is already a known contributor to sensory neuron damage and dysregulation — key mechanisms in cisplatin-induced neuropathy. The gut and nervous system are linked via the gut-brain axis (11), which includes immune pathways, neuroendocrine signalling, microbial metabolites (e.g., short-chain fatty acids). Disruption of the gut microbiome and increased microbial translocation can alter pain signalling pathways, enhance neuronal excitability, worsen or prolong neuropathic symptoms (16).

Several studies have explored molecules with potential protective effects against cisplatin-induced intestinal injury, which could, in turn, lead to a reduction in the overall systemic inflammatory response and consequently help alleviate neuropathic symptoms. However, this potential link has not been further demonstrated in the studies discussed. A 2022 mouse study showed that vitamin D_3_ reduced oxidative stress, limited the accumulation of reactive oxygen species (ROS) and malondialdehyde (MDA), and decreased intestinal inflammation. It also protected the intestine by inhibiting ferroptosis, reducing iron accumulation, and restoring the expression of GPX4 and DHODH (17). Similarly, epigallocatechin gallate (EGCG), the main active compound in green tea, may help preserve the intestinal barrier and reduce bacterial translocation during cisplatin treatment. Although tested only in mice, pretreatment with EGCG significantly alleviated intestinal damage, suggesting a potential protective role (18).

Preclinical studies already show that antibiotics or probiotics that modulate the gut microbiome can reduce chemotherapy-induced neuropathic pain (19). Although human data are still limited, there is growing interest in targeting the gut microbiota as a strategy to prevent or treat CIPN. A specific ex vivo analysis showed that in mice affected by CIPN (induced by paclitaxel), the administration of probiotics increased the expression of opioid and cannabinoid receptors in the spinal cord and influenced the concentration of pro-inflammatory cytokines in the serum (Tnf-α and IL1β and IL6) (11, 19).

Based on our analysis, we observed that elevated sCD14 levels were associated with sensory neuropathy, whereas higher HMGB1 levels correlated with autonomic neuropathy. These findings allow us to hypothesize distinct underlying mechanisms for these biomarkers in chemotherapy-related neurotoxicity. sCD14, as a marker of monocyte and macrophage activation, may reflect peripheral immune activation that contributes to axonal injury in sensory fibers, thereby promoting motor and sensory neuropathies. In contrast, HMGB1 acts as a damage-associated molecular pattern (DAMP) that can activate glial cells within the central nervous system (CNS) via receptors such as RAGE and TLR4. Through these pathways, HMGB1 may induce neuroinflammatory processes in central autonomic regulatory regions, such as the hypothalamus and brainstem, potentially leading to autonomic dysfunction rather than predominantly peripheral nerve injury. Taken together, our data support the hypothesis that sCD14 reflects peripheral neuroinflammation, whereas HMGB1 may be more closely linked to central mechanisms underlying autonomic neuropathy. Importantly, growing evidence suggests that the activation of central immune cells, such as microglia, is not only driven by intrinsic CNS signals but can also be modulated by peripheral factors, including those originating from the gut. Microglia, the immune cells of the central nervous system (CNS), play a key role in both the onset and continuation of chronic pain. These cells respond to signals from the CNS and are also influenced by signals from the gut. Recent studies, both in animals and humans, suggest that interactions between the gut microbiome and microglia contribute to the development of chronic pain. Approaches that alter or restore the gut microbiome have been found to reduce microglial activation and relieve inflammation-related symptoms (20).

Since demonstrating correlation alone is insufficient to justify interventional measures, studies that establish a causal relationship between GMT biomarkers and CIPN are necessary. A logical next step would be to initiate a longitudinal prospective cohort study, allowing for the regular measurement of GMT biomarkers and CIPN symptomatology at multiple time points. This approach would overcome the limitation of the current study, which included only a single measurement at the first post-treatment follow-up. An alternative approach would be to conduct an interventional randomized controlled trial aiming to modulate GMT biomarker levels through the administration of probiotics, prebiotics, or antibiotics, while monitoring changes in CIPN symptomatology. In preclinical mouse models of cisplatin-induced neuropathic pain, fecal microbiota transplantation (FMT) alleviated pain symptoms by suppressing neuroinflammation and oxidative stress, decreasing pro-inflammatory cytokines IL-6 and TNFα while increasing the anti-inflammatory cytokine IL- 10 (21). Interdisciplinary collaboration would also be valuable, utilizing animal models to investigate in detail how GMT biomarkers affect neuronal cells and inflammatory pathways associated with neuropathy.

An important consideration when interpreting our findings is the presence of wide confidence intervals for several key associations, reflecting limited precision of the estimated effect sizes. Although these associations reached statistical significance, the wide intervals indicate considerable uncertainty regarding the true magnitude of the effects. This is likely driven by the relatively small sample size within treatment subgroups, including the radiotherapy (n=14) and combined chemoradiotherapy (n=9) cohorts, as well as the inherent variability of long-term survivorship populations. Such variability may also reflect underlying biological heterogeneity among cancer survivors. Therefore, these findings should be interpreted as exploratory and hypothesis-generating rather than as definitive evidence of clinically robust effects.

Additionally, neuropathy was assessed using a patient-reported outcome measure (EORTC QLQ-CIPN20), which, although validated, is inherently subjective and may be affected by reporting bias. The use of self-reported CIPN questionnaires introduces subjectivity, potentially limiting the accuracy of neuropathy assessment. The absence of objective assessments such as nerve conduction studies (NCS) or quantitative sensory testing (QST) limits the ability to precisely characterize the severity and type of neuropathy. Future studies integrating both patient-reported outcomes and objective neurophysiological measures would provide a more comprehensive evaluation of CIPN and strengthen the clinical interpretation of these findings.

Another potential limitation is confounding bias, as GMT biomarker levels may be influenced by other variables such as diet, antibiotic and probiotic use, body mass index, metabolic conditions including diabetes, alcohol consumption, or acute infections at the time of sample collection. These variables were not systematically collected or controlled for in the present study, which may have influenced the observed associations.

Moreover, the biomarkers assessed, particularly LPS, lack specificity as indicators of GMT. To improve specificity, detection of bacterial DNA in plasma or serum has been proposed as a more precise alternative. However, implementation of such approaches would require larger sample volumes and a substantially larger cohort to allow for robust statistical validation.

## Conclusion

CIPN may be exacerbated by GMT, possibly due to chemotherapy- and radiotherapy-induced gut barrier damage. This may lead to systemic inflammation and immune activation, which may contribute to neurotoxicity and pain. Understanding this link may open avenues for gut-targeted therapies (e.g., probiotics, prebiotics, or anti-inflammatory agents), dietary modifications, or fecal microbiota transplantation to mitigate neuropathy in cancer patients. Our analysis revealed a positive correlation between increased sCD14 levels and the severity of neuropathy in GCT survivors. Moreover, we observed higher levels of sCD14 in patients undergoing chemotherapy compared to the observational group. Our findings suggest that, in addition to sCD14, HMGB1 may serve as a promising biomarker of autonomic dysfunction, particularly in patients who underwent combined oncological therapy. Indeed, modulation of the microbiome may, in the future, become not only an integral component of cancer prevention and treatment strategies, but also a potential approach to mitigating late-onset treatment-related toxicities, which could have a substantial impact on the quality of life of cancer survivors.

## Data Availability

The raw data supporting the conclusions of this article will be made available by the authors, without undue reservation.
